# Isolation and Identification of Bacterial Strains Colonizing the Surface of Biodegradable Polymers

**DOI:** 10.3390/microorganisms13030609

**Published:** 2025-03-06

**Authors:** Roberta Esposito, Serena Federico, Amalia Amato, Thomas Viel, Davide Caramiello, Alberto Macina, Marco Miralto, Luca Ambrosino, Maria Luisa Chiusano, Mariacristina Cocca, Loredana Manfra, Giovanni Libralato, Valerio Zupo, Maria Costantini

**Affiliations:** 1 Department of Ecosustainable Marine Biotechnology, Stazione Zoologica Anton Dohrn, Villa Comunale, 80121 Naples, Italy; roberta.esposito@szn.it (R.E.); serena.federico@szn.it (S.F.); amalia.amato@szn.it (A.A.); thomas.viel7@gmail.com (T.V.); loredana.manfra@isprambiente.it (L.M.); giovanni.libralato@unina.it (G.L.); 2 Department of Biology, University of Naples Federico II, Via Cinthia 26, 80126 Napoli, Italy; 3 Institute of Polymers, Composites and Biomaterials, National Research Council of Italy, Via Campi Flegri, 34, Pozzuoli, 80078 Naples, Italy; mariacristina.cocca@ipcb.cnr.it; 4 Department of Marine Animal Conservation and Public Engagement, Stazione Zoologica Anton Dohrn, Villa Comunale, 1, 80121 Naples, Italy; davide.caramiello@szn.it (D.C.); alberto.macina@szn.it (A.M.); 5 Department of Research Infrastructures for Marine Biological Resources, Stazione Zoologica Anton Dohrn, Villa Comunale, 80121 Napoli, Italy; marco.miralto@szn.it (M.M.); luca.ambrosino@szn.it (L.A.); mcosta@szn.it (M.L.C.); 6 Institute for Environmental Protection and Research (ISPRA), Via Vitaliano Brancati 48, 00144 Rome, Italy; 7 Department of Ecosustainable Marine Biotechnology, Stazione Zoologica Anton Dohrn, Ischia Marine Centre, Ischia, 80077 Naples, Italy

**Keywords:** biodegradable plastics, 16S rRNA gene, mesocosm, microorganisms, molecular identification

## Abstract

Plastics play a key role in every sector of the economy, being used in the manufacturing of products in the fields of health, food packaging, and agriculture. Their mismanagement poses a serious threat to ecosystems and, in general, to human life. For this reason, particular attention has been paid in the last decade to the use of biodegradable polymers (BPs) as an alternative to classic plastics. In this study, we aimed to identify bacterial strains able to colonize the surface of five BPs: poly(butylene succinate) (PBS), poly(butylene succinate-co-butylene adipate) (PBSA), poly(ε-caprolactone), (PCL), poly(3-hydroxybutyrate) (PHB), and poly(lactic acid) (PLA). For this experiment, mesocosms were designed ad hoc to mimic the conditions in which the polymers can be found in marine environments: i. suspended in the water column; ii. laying over gravel; and iii. under gravel. Four bacterial samples were taken (3, 4, 10, and 12 months from the start of the experiment) from five BPs incubated in the above-mentioned three conditions. Our results demonstrated that bacteria belonging to the *Proteobacteria*, *Actinobacteria*, *Firmicutes*, *Bacillota*, *Bacteroidota*, and *Cyanobacteria* phyla were the most frequent colonizers of the surfaces of the five polymers under analysis, and could be responsible for their degradation, resulting in the evolution of strategies to degrade plastics through the secretion of specific enzymes.

## 1. Introduction

Synthetic polymers have been widely used in the last 50 years, thanks to their low cost, light weight, and durability [1], for several applications [2], including the production of packaging materials for food and beverages, the transport of single-use products [3], biomedical items, and tissue engineering [4]. However, the incorrect waste management of plastic products at the end of life and their slow degradation represent a serious environmental issue, considering that around 8 million tons of plastic are estimated to end up in aquatic environments every year [5] and that microplastics have been found in a range of organisms, including those consumed by humans [6,7]. Consequently, attention must be paid to the whole life cycle of any product, from its design to its disposal, to maximize the efficient use of resources. For these reasons, nowadays, the use of biodegradable polymers represents a more eco-friendly opportunity, mitigating environmental impacts compared to the use of synthetic plastic polymers [8,9,10]. Unlike conventional plastics, deriving from fossil fuels, bioplastics are commonly produced from renewable sources such as plants, animals, and microorganisms, and therefore, they are usually biodegradable [11] if correctly managed at the end of life. BPs can be bio-based, i.e., produced from natural sources such as thermoplastic starch (TPS), poly(hydroxyalkanoates) (PHAs), and PLA, or fossil-based, i.e., produced from fossil-based resources such as PCL and poly(butylene adipate terephthalate) (PBAT). In the time frame of 1996–2001, the quantity of biodegradable polymers in use increased from about 14 million Kg to about 60 million Kg, following increases in the sectors where they are employed, from packaging materials to personal hygiene products, as well as toys, mulch films, and other applications [12]. The use of bioplastics contributes to the mitigation of greenhouse gases, with only 0.49 kg of CO_2_ emitted by the production of 1 kg of resin, as opposed to the 2–3 kg of CO_2_ produced by plastics of petrochemical origin [13]. As reported by European Bioplastics (https://www.european-bioplastics.org/; 30 January 2024), biodegradation refers to the chemical process triggered by microorganisms naturally present in the environment that are able to convert various materials into nutrients, mainly depending on the condition of the surrounding environment and the nature of the material. In this context, the phases of the biodegradation of these polymers may undergo variations and alterations according to environmental conditions [14,15]. BPs are used for compostable products to be included in organic recycling, in line with the requirements of the European Packaging and Packaging Waste Directive 94/62/EC. Too little is currently known about the long-term effects on the environment caused by the use of BPs due to poor knowledge of the fate of biodegradable plastics with respect to synthetic ones [16], and they could be harmful to ecosystems if incorrectly disposed [15,17]. Therefore, the use of biodegradable polymers makes such products ecologically acceptable, but attention must be paid to their production, consumption, and removal [18].

Here, we focused on the identification of bacteria growing on the biodegradable polymer surface, assuming that they could be responsible for biodegradation, without consideration of their relative abundance. In fact, microorganisms are able to attach to the surface of polymers and degrade them by secreting enzymes, thereby obtaining energy for their growth [19,20]. Through these mechanisms, polymers can be transformed into compounds characterized by lower molecular weights, such as monomers and oligomers. In this study, we isolated and identified bacterial strains that colonized five biodegradable polymers (BPs) widely used in several fields: polybutylene succinate (PBS), polybutylene succinate-cobutylene adipate (PBSA), polycaprolactone (PCL), poly-3-hydroxybutyrates (PHB), and polylactic acid (PLA).

Much data have been reported on the degradation process of these biodegradable polymers. MPs can promote the development of microbial biofilms on their surface [21], known as the plastisphere microbiota [22,23,24]. Microorganisms can directly interact with the plastic surface and break down or transform plastic compounds [25]. Kim et al. [26] reported that PBS fishing gear was degraded into carbon in 180 days, exhibiting a characteristic molecular weight decrease indicating bulk erosion, but there was no change in crystallinity or thermal properties. This degradation process proceeded through surface abrasion with the metabolic utilization of hydrolysis products by microorganisms. Biological studies showed that the *Rhodococcus* genus was the dominant microbe for PBS biodegradation, using a lipase enzyme with a high biodegradability of 48% over 10 days. PCL degradation is influenced by the combined effect of factors such as the presence of bacteria, salinity, and external forces in the sea [27]. Microorganisms known to degrade PHB include *Bacillus* spp., *Microbulbifer* spp., *Ralstonia eutropha*, and the genus *Streptomyces* through PHB depolymerase [28]. *B. infantis*, among the *Bacillus* spp., has been found in various environments, such as soil and sea, and has the ability to degrade azo dyes that are difficult to biodegrade. PLA was presented as a substrate to stimulate the production of protease [29]. *Pseudomonas* [30], *Paenibacillus* [31], and *Aspergillus* [32] represent the most widely present potential plastic-degrading microorganisms in the plastisphere [33]. In particular, Pseudomonas spp. and Bacillus spp. were isolated as plastic-degrading bacteria from marine environments, such as offshore and deep oceanic waters [34].

Amid this context, in this work mesocosms were built ad hoc in order to mimic the possible conditions in which polymers can be found in marine environments: suspended in the water column, laying over gravel, or under gravel. The process of the colonization of plastic surfaces was followed for one year in order to follow the seasonal plasticity of the bacteria to demonstrate how they changed according to the nature of the employed biopolymers, and also to consider tank parameters, like temperature, light, and other physicochemical parameters, as putative causal factors. In particular, molecular identification was performed on bacteria grown on the five BPs under analysis through the amplification of the *16S rDNA* gene.

## 2. Materials and Methods

### 2.1. Mesocosms

**Figure 1:**
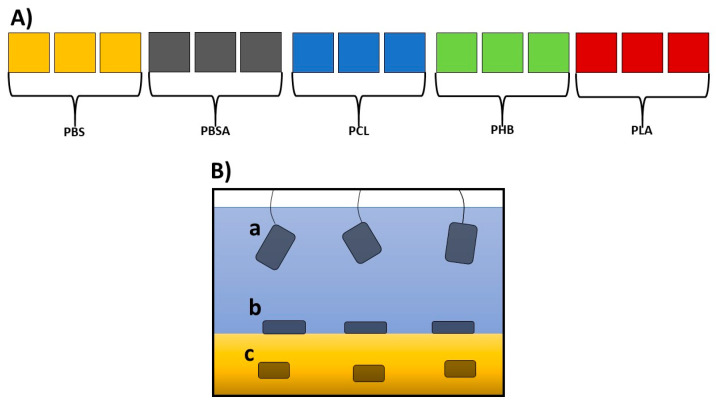
(**A**) Schematic overview of the experimental mesocosms: fifteen experimental tanks (length: 48.5 cm; width: 36.6 cm; height: 50 cm; volume 55 L), three for each of the five polymers under analysis. (**B**) Enlargement of a tank, showing the three experimental conditions where the polymers were placed: (**a**) suspended in sea water, immersed about 10 cm from the surface of the water; (**b**) laying over gravel; (**c**) under gravel.

A set of fifteen independent, closed circulation tanks with a volume of 55 L (three for each of the five polymers; Figure 1A; see also Figure S1) was devised in the facility for the Maintenance of Marine Organisms of the Stazione Zoologica Anton Dohrn (Naples).

The bottom of each tank was covered with natural gravel (coralline sand, grain size 0.4–0.8, Arena Silex, Manufacturas Gre, S.A., Biscay, Spain), and each tank was filled with 50 L of filtered seawater (filtered with VakuumFiltration from TPP Techno Plastic Products AG with PES membrane, 0.22 µm pore size) collected from the Gulf of Naples. The water was continuously pumped (Micra 400 L/h, SICCE, Pozzoleone, VI, Italy) into a filtration compartment containing porous ceramic rings, a 20 PPI non-biodegradable synthetic sponge (Aquarialand, Torino, Italy), and perlon wool [17,35]. To avoid any salinity variation, the evaporation was balanced with periodic additions of distilled water. This system assured a constant temperature of 18 °C, a salinity of 37–38 PSU, a pH close to 8.2, and low concentrations of the main nutrients (NH_4_, NO2, NO3, and PO_4_) (see Tables S1 and S2). After each addition, the salinity was checked by a refractometer (Sper Scientific, Scottsdale, Arizona). Polymers were produced according to the procedures indicated by Viel et al. [15]. Rectangular sheets of the five BPs (PLA, PCL, PHB, PBSA, and PBS) sized 15 cm × 5 cm with a thickness of 1 mm were introduced in each tank in specific filter bags for aquaria in triplicate for each of the three conditions indicated in Figure 1B.

### 2.2. Bacterial Strain Identification

#### 2.2.1. Analysis of Film Surface

Surface morphologies of the samples were investigated by SEM analysis, performed with an FEI Quanta 200 FEG environmental scanning electron microscope (ESEM) (Eindhoven, The Netherlands) under high vacuum using an accelerating voltage range of 30 kV and a large field detector (LFD). Small pieces of BPs before and after 1 year of immersion in the water column were cut and placed without any purification on SEM stubs. Film surfaces were coated with a nanometric layer of Au and Pd alloy by means of a sputtering device (MED 020, Bal-Tec AG, Tucson, AZ, USA).

#### 2.2.2. Bacterial Growth and Strain Isolation

For the isolation of bacterial strains, Zobell Marine Agar (Marine Agar 2216, HIMEDIA, Laboratories GmbH, Odenwaldstr. 18 A, 64397 Modautal, Germany) was used (Table S3). The media were prepared according to the manufacturer’s instructions, sterilized, and then poured into sterile Petri dishes. Bacteria were picked from the surfaces of the sheets four times, i.e., 3, 4, 10, and 12 months (corresponding to October, November, May, and July, in order to identify bacterial strains colonizing the BP surfaces in different seasons, characterized by different sea water temperatures) after deployment, in order to detect the first settlements of bacterial strains and the final assemblages of bacterial communities. Microbial film was scratched from the plastic surface with a sterile microbiological loop and the bacteria were spread on the surface of the Zobell Marine Agar contained in the Petri dishes to obtain a pure culture of bacteria, so a single cell occupied an isolated portion of the agar surface. The samples were incubated in a thermostatic chamber at 18 °C under a 12 h:12 h light–dark photoperiod until bacterial colonies had grown (about 10 days) [36,37]. The bacterial strains were identified by a molecular approach without considering the relative abundance.

#### 2.2.3. DNA Extraction and Molecular Identification

DNA was extracted from the bacterial colonies using a lysis PCR-GO Fast-G Kit (#BombaMIXFastGReen, Spinoff DNATech, University of Naples, Naples, Italy). The kit is specifically designed for use with PCR, performed directly from bacterial or fungal colonies. The DNA extraction was performed according to the manufacturer’s instructions by simply adding a lysis buffer. The quality of the extracted DNA was evaluated using a NanoDrop spectrophotometer (ND1000 UVVIS Spectrophotometer; NanoDrop Technologies, Wilmington, DE, USA) by checking the concentration and the purity at the 260/280 and 260/230 ratios. A PCR amplification of prokaryotic 16S rDNA was then performed using 12.5 µL of 2× PCR Mix-Go Hot Start according to the manufacturer’s instructions with 27F-1385R primers [38] under the following conditions: initial denaturation at 95 °C for 5 min; denaturation at 95 °C for 45 s; annealing step at 55 °C for 45 s; extension at 72 °C for 1.30 min; final extension at 72 °C for 5 min. An agarose gel (1% electrophoresis in 40 mM Tris–acetate, 1 mM EDTA, pH 8.0, TAE buffer solution containing a mixture of Tris base, acetic acid, and EDTA) was used for the separation of the PCR products. The extraction of the fragments from the gel was performed with an QIAquick Gel Extraction Kit (Qiagen, Hilden, Germany) according to the manufacturer’s instructions. The quantity of DNA was evaluated using the above-mentioned NanoDrop spectrophotometer. The sequences of amplicons were obtained with an Applied Biosystems (Life Technologies, Carlsbad, CA, USA) 3730 Analyzer (48 capillaries). The similarity of the sequences of the amplified fragments were checked using a nucleotide BLAST (Basic Local Alignment Search Tool, https://blast.ncbi.nlm.nih.gov/Blast.cgi; 30 January 2024 [39]), using covering >50% and identity > 95% for the assignment of species to a given taxon.

### 2.3. MCA Analysis

The data collected as indicated above were initially organized in a presence/absence matrix, where detected/not detected (1/0) indices for each bacterium on each sample and plastic condition were shown. To analyse the relationships among these categorical variables, a Multi-Correspondence Analysis (MCA) was performed. Data were thus one-hot encoded for this purpose, creating a binary column for the two possible values of each variable. The results of these analyses were reported using a biplot technique in order to show on the same factorial plane both the bacteria strains and those plastic variables encoding presence. The first two components resulting from the MCA were shown on the cartesian plane. The MCA was conducted using a custom Python 3 3.13.2 [40] script, scikit-learn [41], and the prince [42] library. Plots were obtained using the seaborn library [43]. A chi-squared test was performed on the frequency data on the observed presence of each bacterium across all months and conditions by setting (k − 1) degrees of freedom, where k is the number of categories.

## 3. Results

**Figure 2:**
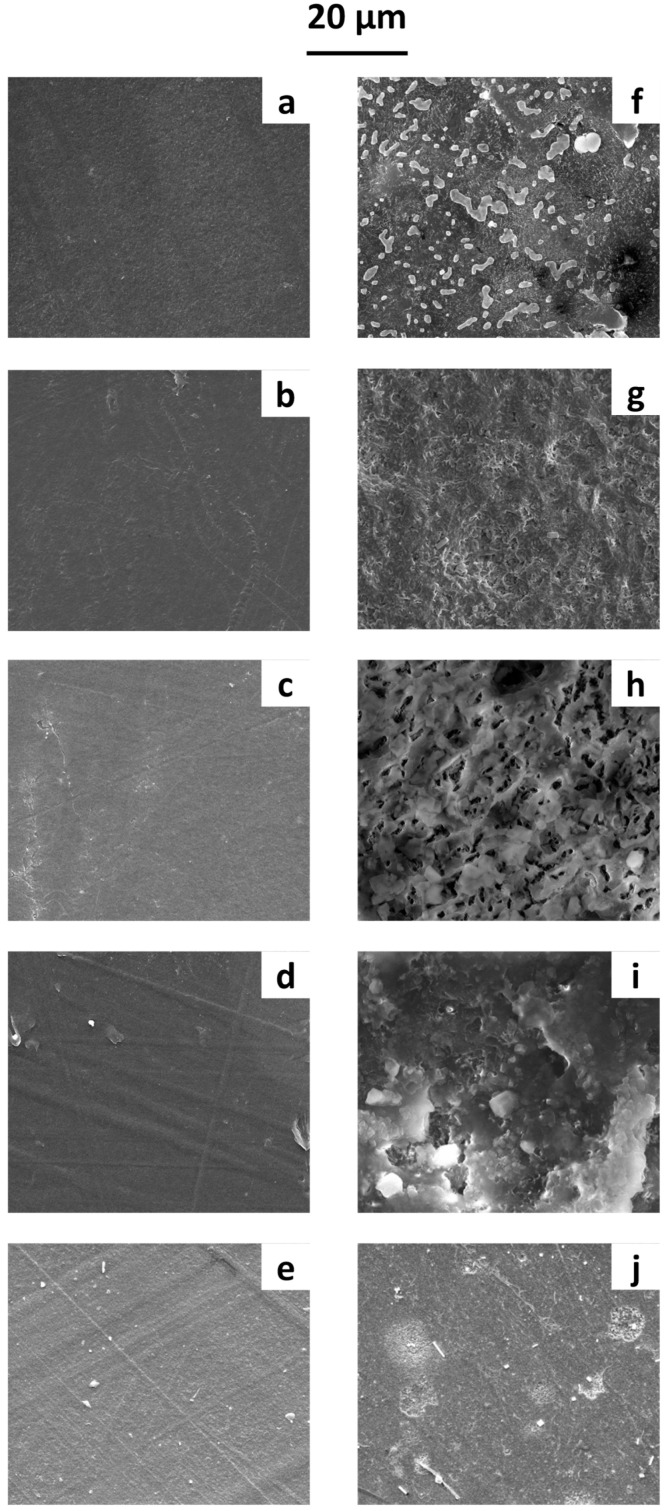
SEM micrographs of film surfaces before immersion in water: (**a**) PBS, (**b**) PBSA, (**c**) PCL, (**d**) PHB, and (**e**) PLA films. After 1 year of immersion in water: (**f**) PBS, (**g**) PBSA, (**h**) PCL, (**i**) PHB, and (**j**) PLA films.

Thin films were prepared using different raw polymers: poly(butylene succinate (PBS), poly(butylene succinate-co-adipate) (PBSA), polycaprolactone (PCL), polyhydroxy butyrate (PHB), and polylactic acid (PLA). The materials were processed to obtain films approximately 300 µm thick, following the procedure described in De Falco et al. [44]. SEM micrographs before and after 1 year of immersion in water are reported in Figure 2.

Before immersion (Figure 2a–e), the film surfaces were smooth and lacked significant roughness. After one year in water, the SEM micrographs (Figure 2f–j) allowed us to examine the microbial growth on the polymeric surface. Biofilm formation was observed on all films, with SEM micrographs clearly showing various cell shapes and extracellular adhesive structures, likely exopolysaccharides and proteinaceous adhesins. Differences in microbial growth patterns were evident across the polymeric surfaces—likely influenced by the surface properties of the polymers, leading to distinct biofilm formation dynamics.

Several bacteria strains were identified on the surface of the five BPs analysed, and were reported as present/absent without considering their abundance. The first samples were taken three months after the start of the experiment, because no bacteria were detected before three months. All the bacterial strains isolated on the surfaces of the five BPs in the three deployment conditions (suspended, over gravel, or under gravel), considering the four sampling points, were analysed according to the results of the MCA to describe their developmental trends.

### 3.1. PBS

No bacteria were isolated in the third month (October) from the PBS polymer suspended in seawater. *Bacillus* sp. (Phylum *Firmicutes*), *Marinobacterium rhizophilum*, and *Pseudomonas* sp. (belonging to the Phylum *Proteobacteria*) were isolated in the third month from PBS positioned over the gravel (Table 1).

The *Rhodobacteriaceae* and *Pseudoalteromonadacea* families (belonging to the Phylum *Proteobacteria*) were isolated in the third month from PBS positioned under gravel. Similarly, no bacterial strains were found at the second collection point (forth month) on PBS positioned over gravel. Only one bacterial strain, *Vibrio variabilis* (Phylum *Proteobacteria*), was isolated in the fourth month from PBS suspended in seawater. In contrast, *Rhodobacteriacea* and *Halomonadaceae* (both belonging to the Phylum *Proteobacteria*) were detected on PBS positioned under gravel. In the tenth month, *Pontibacterium* sp. and *Pseudoalteromas* sp. (Phylum *Proteobacteria*) were isolated from suspended PBS. *Calothrix* sp. (belonging to the Phylum *Cyanobacteria*), along with *Marinobacter* sp., *Pseudoalteromonas citrea*, and *Vibrio* sp. (Phylum *Proteobacteria*), were identified on PBS positioned over gravel. Differently, *Neptuniibacter* sp. and *Paenihalocynthiibacter styelae* were isolated from PBS positioned under gravel. In the twelfth month, the largest number of bacterial strains was detected. In particular, *Halomonas* sp., *Paenihalocynthiibacter styelae*, *Ruegeria* sp., and *Vibrio caribbeanicus* (all belonging all to the Phylum *Proteobacteria*), and one family of *Microbacteriaceae* (hylum Actinobacteria) were detected on PBS suspended in the water column. Three species of bacteria, *Marinobacterium jannaschii*, *Paenihalocynthiibacter styelae*, and *Vibrio caribbeanicus* (Phylum *Proteobacteria*), were detected in the tenth month on PBS positioned over gravel. Only one strain was identified in the twelfth month at the species level (*Marinobacterium jannaschii*), and two strains were identified at the family level (*Alteromonadaceae* and *Rhodobacteracea*) on PBS positioned under gravel.

### 3.2. PBSA

Only the family of *Bacillaceae* (Phylum *Firmicutes*) was found in the third month on PBSA suspended in seawater. The families *Alteromonadaceae*, *Rhodobacteraceae*, *Pseudomonadaceae*, and *Vibrionaceae* (all belonging to the phylum *Proteobacteria*) were isolated from PBSA positioned over gravel (Table 2).

Three bacterial families, identified as *Alteromonadaceae*, *Rhodobacteriaceae*, and *Pseudomonadaceae*, and one bacterial strain identified at the species level as *Rosebium aggregatum* were isolated on the polymer positioned under gravel. The species *Vibrio alginolyticus* was identified in the fourth month on PBSA suspended in water, while the family Vibrionaceae was isolated from the film over the gravel. *Cyanobium* sp. (Phylum *Cyanobacteria*), *Halomonas* sp., *Ruegeria* sp., and *Tritonnibacter scottomollicae* (all belonging to the phylum *Proteobacteria*) were found in the tenth month on the polymer suspended in the water. One species of *Paenihalocynthiibacter styelae* and one family identified as *Pseudoalteromonadaceae* were found in the tenth month on PBSA over the gravel. *Pseudoalteromonas citrea* and *Psychrobacter* sp. were isolated in the same month from plastic pieces under gravel. *Paenihalocynthiibacter styelae* (Phylum *Proteobacteria*), *Rathayibacter* sp. (Phylum *Actinobacteria*), and *Tenacibaculum* sp. (Phylum *Bacteroidota*) were identified in the twelfth month on film suspended in seawater. *Bacillaceae*, *Alteromonadaceae*, and *Flavobacteriaceae* families were found on the polymer positioned over the gravel. Differently, *Leisingera aquaemixtae* and *Paenihalocynthiibacter styelae* (Phylum *Proteobacteria*) were identified on the PBSA positioned under gravel.

### 3.3. PCL

The genera *Pseudomonas* and *Sulfitobacter* and the species *Marinobacter algicola* (all belonging to the Phylum *Proteobacteria*), together with the species *Kocuria rosea* (Phylum *Actinobacteria*), were found in the third month on PCL suspended in the water (Table 3).

The genera *Halomonas*, *Marinobacter*, and *Pseudomonas* were isolated in the same month from PCL over the gravel, while *Halopseudomonas*, *Pseudoalteromonas*, *Pseudomonas*, and the species *Alkalihalobacillus algicola* (belonging to the phylum *Firmicutes*) were identified on PCL under gravel. Only one bacterial strain belonging to the genus *Paraglaciecola* was identified in the fourth month on the PCL surface suspended in water. The genera *Bacillus*, *Pseudomonas*, and *Ruegeria*, and the species *Rossellomorea vietnamensis* and *Tritonnibacter scottomollicae* were found on PCL over the gravel. Four genera (*Pseudoalteromonas*, *Pseudomonas*, *Paraglaciecola*, and *Glaciecola*) and three species (*Halopseudomonas gallaciens*, *Marinobacter sediminum*, and *Marinobacter similis*) were isolated from the polymer under gravel. The genus of *Ruegeria* was identified in the tenth month on the plastic suspended in the water. Differently, the phylum *Proteobacteria* and the species *Pseudoalkalibacillus hwajinpoensis* (Phylum *Bacillota*) were isolated from PCL positioned over the gravel. Only one species of *Paenihalocynthiibacter styelae* (Phylum *Proteobacteria*) was found on film under gravel. The genera *Alteromonas* (Phylum *Proteobacteria*) and the genera *Tenacibaculum* (Phylum *Bacteroidota*) were detected in the twelfth month on the PCL surface suspended in the water. *Alteromonas, Photobacterium, Roseovarius* (Phylum *Proteobacteria*), *Bacillus* sp. (Phylum *Firmicutes*), *Magnetococcus* sp., and *Thalassospira* sp. (Phylum *Proteobacteria*) were isolated from the PCL film surface positioned under the gravel.

### 3.4. PHB

The genera *Halomonas* and *Marinobacter* (both belonging to the phylum Proteobacteria) were identified in the third month from the PHB suspended in the water. The species *Pseudoalteromonas prydzensis* and the genus *Pseudomonas* were found on this polymer positioned over the gravel, while the genera *Marinobacter* and *Vibrio* were found on the PHB positioned under the gravel (see Table 4).

No bacterial strains were detected in the fourth month on the polymer suspended in the water. On the contrary, the species *Vibrio corallilyticus* was identified from PHB. The genera *Pseudomonas* and *Vibrio* were found on the polymer surface under gravel. The species *Marinobacter algicola* (Phylum *Proteobacteria*) was found in the tenth month on the plastic suspended in the water and on the plastic positioned over the gravel. In addition, the species *Pseudoalteromonas citrea* was identified on the PHB over the gravel. Three genera (*Entobacter, Pseudoalteromonas, and Thalassospira*) and two species (*Pseudomonas aeruginosa* and *Vibrio alginolyticus*) were found on the PHB positioned under gravel. Three genera (*Labrenzia, Marinobacter*, and *Vibrio*) and one species (*Roseibium aggregatum*) were found at the fourth collection point (the twelfth month) on the PHB film surface suspended in the water. In contrast, the species *Tritonibacter litoralis* (Phylum *Proteobacteria*) was isolated from PHB positioned over the gravel, while the genus *Thalassospira* (also belonging to the phylum *Proteobacteria*) was identified from PHB positioned under the gravel.

### 3.5. PLA

Three species (*Alteromonas australica*, *Idiomarina ioihiensis*, and *Idiomarina ramblicola*) and two genera (*Cyanobium* and *Microbulbifer*) were found in October on PLA suspended in water. Differently, the species *Alteromonas australica* and *Halomonas taenennsis* were identified from PLA positioned over the gravel (as reported in Table 5).

Two genera, *Alteromonas* and *Marinobacter* (belonging to the Phylum Proteobacteria), and one species of *Bacillus aquimaris* (Phylum *Firmicutes*) were isolated from PLA positioned under the gravel. The family of *Alteromonadaceae* and the genus of *Pseudomonas* were found in the fourth month on this polymer suspended in the water. Only the species *Alteromonas macleodii* (Phylum *Proteobacteria*) was isolated from PCL positioned under the gravel. The genera *Glaciecola*, *Paraglaciecola*, and *Psychrobacter* and the family of Rhodobacteracea were identified in the tenth month on PLA suspended in the water. *Halomonas* sp., *Phaeobacyer* sp., *Leisingera* sp., *Pseudoalteromonas* sp., and the species of *Pseudoalteromonas citrea* were isolated from PCL positioned over the gravel and from PCL positioned under gravel. Moreover, the species *Alteromonas australica* and *Pseudoalkalibacillus hwajinpoensis* and the genera *Alteromonas*, *Halomonas*, and *Pseudopelagicola* were found on the PLA suspended in the water. Only one species, *Sutcliffiella horikoshi* (Phylum *Bacillota*), was isolated from PCL positioned over the gravel. The species *Leisingera methylohalidivorans* and *Sulfitobacter dubius* (both belonging to the phylum *Proteobacteria*) and the genera *Ruegeria* and *Tenacibaculum* were identified from the PLA surface positioned under the gravel.

### 3.6. MCA Analysis

**Figure 3:**
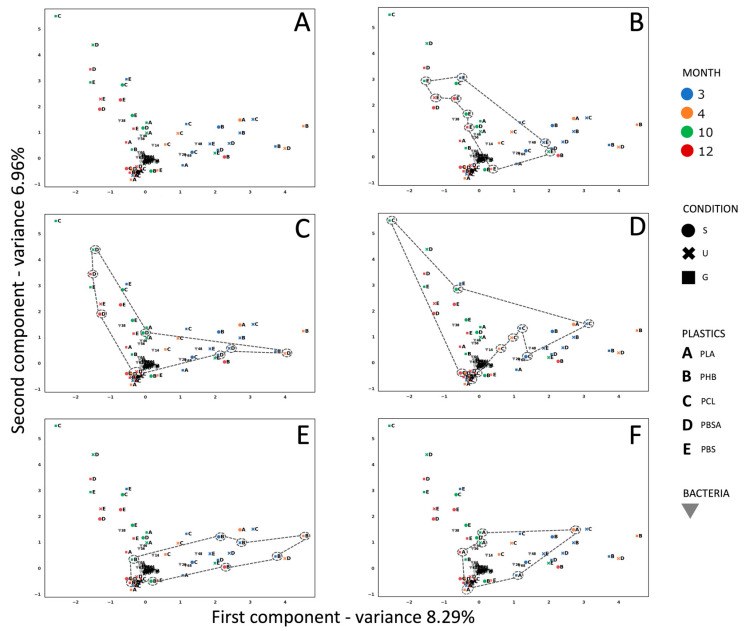
(**A**) MCA analysis showing the correlation between experimental conditions, i.e., different observations related to the polymers in the mesocosm, different positions of the polymers in the mesocosm (S = suspended; U = under gravel; G = gravel), and different months in which the bacterial strains were isolated (3 = October 2021, 4 = November 2021, 10 = May 2022, 12 = July 2022). (**B**) PBS observations are highlighted; (**C**) PBSA observations are highlighted; (**D**) PCL observations are highlighted; (**E**) PHB observations are highlighted; (**F**) PLA observations are highlighted.

This analysis summarizes and allows visualization in a factorial plane of the correlations between the bacterial strains isolated in different months and on different plastics (Figure 3A).

According to the broken-stick model [45], the axes are both significant. A cyclical trend characterizes the space assemblages of all polymers under analysis. More specifically, in the case of PBS (Figure 3B), the cyclical trend started in October (which represents the starting point of the bacterial characterization) in the over-the-gravel condition, before moving to the under-gravel condition in May, the over-gravel condition in November, over the gravel again in July, and then to the suspended condition and further under-the-gravel conditions, leading back to May, with over the gravel. For this polymer, two families of bacteria (*Alteromonadaceae* and *Oceanospirillaceae*) appeared in the third, tenth, and twelfth months, which in turn corresponded to the seasons with the highest temperatures. The presence of the family of *Rhodobacteraceae* was constant over time, regardless of temperature and the degradation of the plastic samples. The *Pseudoalteromonadaceae* family was identified only on film samples collected in the months of October and May, with a temperature of 20 ± 1 °C. For PBSA (Figure 3C), always starting from October in the under-the-gravel gravel condition, the cyclical trend passed through July (under gravel, suspended, and gravel conditions) and then May (under gravel, but in the same month suspended), before arriving again to the under-gravel condition in October. It is noteworthy that the families of *Rhodobacteraceae*, *Flavobacteraceae*, and *Microbacteracea* appeared in October, May, and July with temperatures of 20 °C, 21 °C, and 28 °C, respectively. The cyclical trend of PCL (Figure 3D) started from October with under the gravel, moved to the suspended condition in the same month, then passed to the gravel condition, then to over the gravel and under gravel in July, until May (over the gravel and suspended), before finally coming back to October. For PHB (Figure 3E), the trend in October started from suspended/under-gravel conditions, moved to the gravel condition in November, then to over the gravel in October, suspended in July, then over the gravel again in May, back to the starting point of October. For PLA (Figure 3F), the trend started as over the gravel in October, before moving in the same month to the under-gravel condition; in July the condition was over the gravel, then under the gravel in May, passing to November, when it was suspended, before returning to October. The family of *Alteromonadaceae* was constant over time on this polymer; while the family of *Halomonadaceae* was found in October, May, and July, *Rhodobacteraceae* were found in May and July and *Flavobacteraceae* were found only in the warmest months.

Finally, we retrieved a significant *p*-value of 1.50 E10^−6^ as the result of a chi-squared test performed on the frequency data of the observed presence of each bacterium across all months and conditions.

## 4. Discussion

The presence of bacteria on plastics was described for the first time in 1972 by Carpenter at al. [46] and Carpenter and Smith [47], but more recently, studies have focused on the composition of microbial communities able to colonize plastic surfaces [48] and on the functional optimization of enzymes in order to obtain an economically feasible way to biodegrade plastics [49,50]. Bacteria belonging to *Proteobacteria*, *Actinobacteria*, *Firmicutes*, *Bacillota*, *Bacteroidota*, and *Cyanobacteria* were the most frequent colonizers of microplastic surfaces. Within the Phylum *Proteobacteria*, the families of *Alteromandaceae*, *Pseudomonadaceae*, *Rhodobacteraceae*, and *Halomonadaceae* were identified on all five plastics under analysis. Bacteria belonging to the family *Rhodobacteraceae* are known as “initial colonizers” of different substrates in various marine environments, suggesting a non-specificity for the plastic’s surface [51]. The family *Pseudomonadaceae*, already detected in soil and waste sites [52,53], was also found in association with microplastics [54,55,56,57,58,59]. Our results showed that the *Gammaproteobacteria* family of *Oceanospirillaceae* was present on the surface of PBS, while *Vibrionaceae* were present on PBS, PCL, PBSA, and PHB surfaces. Moreover, we noticed differences in the composition of the bacterial communities that colonized the film surfaces in response to variations in temperature during the seasons herein analysed. In fact, within the phylum of Proteobacteria, the family of *Alteromonadaceae* was present on the surface of all polymeric films in October and July, while in the coldest month analysed (November) it was present only on PLA and PCL. The family of Pseudomonadaceae was abundant in November on PLA, PHB, and PCL, while it was present in October on PHB, PCL, PBSA, and PBS. In the warmest month (July), we found *Rhodobacteraceae*’s family on all film surfaces analysed. Finally, the family of *Halomonadaceae* was found on the surface of PLA, PHB, and PCL in October. These results demonstrate that there is no specificity for the type of condition in which the microplastics were found, but there is a correlation between sea water temperature and bacterial growth. In the last fifteen years, researchers have demonstrated the presence of bacteria belonging to the family *Flavobacteraceae* (*Bacteriodetes* phylum) on PS, (LD)PE, PP, and PET plastics [22,60,61,62,63,64,65,66]. Our results confirmed not only that this family was found in association with the microplastics used—and in particular PLA, PCL, and PBSA—but also that these bacteria appeared on plastic surfaces only in the warmest month analysed (July), showing a good adaption to higher temperatures. As for the class *Microbacteriaceae*, which belongs to the Phylum *Actinobacteria*, it was detected on PBSA and PBS only in the warmest month (July). Besides showing an adaptation to high temperatures, previous research has demonstrated that these bacteria are good candidates for the bioremediation of plastic wastes [67,68]. The phylum of *Cyanobacteria* was detected on PLA, PBSA, and PBS microplastics in October and May. Cyanobacteria are known to colonize different types of plastics, such as PE, PET, and PP [60,63,69,70], but no data are available, to the best of our knowledge, about their presence on biodegradable plastics.

These data confirm the idea that the nature of the employed biopolymers was not the only factor explaining the shifts in biofilm composition. For example, temperature in our experiments was a very important factor influencing the growth of bacteria. For this reason, we think that our results were significant because the bacterial growth on plastic films was detected for one year in order to follow the seasonal plasticity of the bacteria. If we consider the data reported in the literature on the biodegradable polymers analysed, many of the bacteria isolated on the surface of the five BPs analysed in this work are able to synthetize enzymes that are able to degrade plastics. Enzymatic biocatalysis is crucial in the biodegradation of plastics. These enzymes are first adsorbed on the film surface with the help of the surface-binding domain, and then break the chemical bonds of plastic [34]. PBS has been reported as non-biodegradable in marine environments, and until now, few studies have shown PBS-degrading marine microbes. Kimura et al. [71] showed the presence of *Vibrionaceae* (*Vibrio ruber*, *Vibrio rhizosphaerae*, and *Vibrio spartinae*) and *Pseudoalteromonadaceae* on PBS films. Further gene identification indicated genes related to a PBS-degrading enzyme (PBSase) produced by these bacteria. *Terribacillus* sp. JY49 was also identified as a potential bacterial strain that can degrade PBS and other bioplastics [72]. The isolate, identified as a member of the genus *Halopseudomonas*, demonstrated PBSA degradation potential at the salinity levels of seawater. In addition to PBSA, the strain could degrade PCL [73,74], and has been utilized as a model to investigate the biodegradation potential of bacteria and their involved catabolic enzymes [75], during which the *Rhodococcus erythropolis* D4 strain was isolated. Also *Pseudomonas pachastrellae* JCM12285T was identified as a PCL-degrading bacterium through its hydrolytic activity with the hydrolysate 6-hydroxyhexanoic acid (6HH), in addition to a major component of cutin, 16- hydroxyhexadecanoic acid [59]. The *Brevundimonas* sp. strain MRL-AN1 was isolated as a PCL-degrading bacterium [76]. PHB degradation was performed by *Bacillus infantis* through PHB depolymerase [28]. Two isolates of potent PLA-degrading bacteria were selected and identified as *Stenotrophomonas pavanii* and *Pseudomonas geniculate* [29]. The bacterium *Roseibium aggregatum* ZY-1 was able to colonize poly(butylene adipate-co-terephthalate) PLA (PBAT-PLA) films and degrade this polymer using the action of various enzymes, such as PETase, carboxylesterases, arylesterase (PpEst), and genes like pobA, pcaBCDFGHIJKT, dcaAEIJK, and paaGHJ, which are involved in PBAT degradation [77]. *Halomonas* sp. showed significant degradation capabilities against poly (ethylene terephthalate) (PET) [78]. *Bacillus* species were able to degrade low-density polyethylene (PE) [79]. Comparative data on PCL, PBSA, PLA, and PBS in film and powder forms showed that their biodegradation strictly depended on the shape of the polymer, according to which the plastic was easily or slowly degraded [80,81,82]; at the early stage of the biodegradation, biodegradable plastics in powder form with a larger surface area were biodegraded faster than the same plastics in film form. At the late stage of biodegradation, the biodegradation rates of PCL and PBSA, considered easily biodegradable plastics, were independent of the form of the samples, while the effect was constant for PLLA and PBS. The biodegradation of PCL, PHB, PLA, and PBS was also studied by Hosni et al. [83], where these BPs were compared in soil and compost over a period of more than 10 months at 25 °C, 37 °C, and 50 °C. Degradation rates varied between the polymers and incubation temperatures, but PCL showed the fastest degradation rate under all conditions. Furthermore, various fungal strains on the polymer surfaces were identified, such as *Aspergillus fumigatus*, which was mostly found at 25 °C and 37 °C, and *Thermomyces lanuginosus*, which was associated with PCL and isolated at 50 °C.

Nowadays, the presence in the environment and the actual fate of biodegradable polymers are critical issues in research aimed at facilitating their disposal at the end of their use in order to reduce their environmental impact. In particular, their degradation, closely related to their structure, properties, and behaviour, is being extensively investigated. Their effects on plastic degradation and the bioremediation potential of bacteria make these microorganisms biotechnologically important because they permit the development of potent tools to reduce the presence of harmful plastics in ecosystems. Here, we demonstrated their potential role in the transformation of biodegradable polymers, and more in general of plastic wastes, and their easy adaptation to higher temperatures. Noteworthily, it is important to state the limitations of the culturable strategy employed, as other microorganisms like viruses, archaea, eukaryotes [84], and non-culturable bacteria were not characterized, and could have participated in the biofilm structure.

Furthermore, the bacteria isolated in the present work on each of the biodegradable polymers could represent specific consortia potentially able to secrete specific degrading enzymes (see Figure S2). Further research is needed to examine potential relevant degradation mechanisms, such as those involving key enzymes [18,85]. An important issue regarding the degradation of plastics is represented by the use of microbial consortia, which are able to degrade complex compounds, such as plastic materials; this seems to be more advantageous compared to isolated bacteria [86,87]. In fact, it was demonstrated that consortia were more adaptable and stable, representing a useful catalytic environment for enzymes involved in biodegradation pathways, such as hydrolases, cutinases, carboxylesterases, lipases, esterase, peroxidase, laccase, manganese superoxide dismutase, and alkane hydroxylase.

This aspect is very important if we consider the increase in the production and accumulation of recalcitrant materials, such as plastics, and the need to produce new, sustainable, and challenging strategies for plastic biodegradation with the aim of reducing environmental pollution [25]. The changes in community structure that occurred during the seasons pave the way for the discovery of specific decomposers in a stable consortium. The current research on complex-compound-degrading microbial consortia, helped by advances in metabolic engineering and synthetic biology (involving genetic modification, genetic manipulation, gene cloning, and recombinant DNA technology) represents a new approach for the efficient utilization of complex constructions of microbial consortia with strong degradation potential. In fact, microbial cells can be genetically engineered to degrade plastic contaminants in the environment more effectively [88]. In conclusion, the microbial and enzymatic degradation of plastics is a promising strategy for the depolymerization of waste plastics and to covert waste plastics into higher-value bioproducts, making them more environmentally friendly and sustainable, and offering a green alternative to plastic recycling.

## Figures and Tables

**Table 1:** Species, month (3 = third month, October; 4 = forth month, November; 10 = tenth month, May; 12 = twelfth month, July), condition (S: suspended; G: over gravel; U: under gravel), accession number, and percentage of identity are reported for PBS. ND indicates strains for which the species could not be defined.

Species	Month (Condition)	Accession	Identity (%)
*Calothrix* sp.	10 (G)	KM019977.1	98.7
*Halomonas* sp.	12 (S)	MT653353.1	88.6
*Labrenzia* sp.	4 (U)	KY770164.1	95.8
*Marinobacter jannaschii*	12 (G, U)	NR_113757.1	97.8
*Marinobacter* sp.	10 (G)	MF382075.1	99.6
*Marinobacterium rhizophilium*	3 (G)	GQ245896.1	99.4
*Marinobacterium* sp.	12 (U)	PQ278257.1	97.2
*Microbacterium* sp.	12 (S)	KX496339.1	97.9
ND	4 (G)		
*Paenihalocynthiibacter stylae*	10 (U), 12 (S, G)	NR_181360.1	99.2
*Pontibacterium sinense*	10 (S, U)	MZ222395.1	99.6
*Pseudoalteromonas citrea*	10 (S)	KX453254.1	97.9
*Pseudoalteromonas* sp.	3 (U)	OP209750.1	99.4
*Pseudomonas citrea*	10 (G)	KX453254.1	98.1
*Pseudonomas* sp.	3 (G)	MT180478.1	90
*Rossellomorea aquimaris*	3 (G)	MH261129.1	99.4
*Ruegeria* sp.	3 (U), 12 (S, U)	KY770077.1	100
*Salinicola* sp.	4 (U)	KC834569.1	99.8
*Vibrio caribbeanicus*	12 (S, G)	KP329557.1	100
*Vibrio* sp.	10 (G)	FJ357697.1	93.7
*Vibrio variabilis*	3 (S), 4 (S)	MT269597.1	97.9

**Table 2:** Species, month (3 = third month, October; 4 = forth month, November; 10 = tenth month, May; 12 = twelfth month, July), condition (S: suspended; G: over gravel; U: under gravel), accession number, and percentage of identity are reported for PBSA.

Species	Month (Condition)	Accession	Identity (%)
*Alkalihalobacillus* sp.	3 (S)	KP297330.1	98.6
*Alteromonas confluentis*	12 (G)	OP583651.1	99.44
*Bacillus* sp.	12 (G)	KF966199.1	78.1
*Cyanobium* sp.	10 (S)	KU951691.1	95.2
*Halomonas* sp.	10 (S)	EU868853.1	99.2
*Labrenzia* sp.	3 (U)	MG025898.1	98.2
*Leisingera aquaemixtae*	12 (U)	MH283824.1	97.4
*Marinobacter* sp.	3 (U)	LT600659.1	99.2
*Marinobacterium* sp.	3 (G)	PQ278257.1	99.4
*Marinovum* sp.	3 (U)	MT673143.1	97.1
*Oceanicola* sp.	3 (G)	CP040932.1	97.4
*Paenihalocynthiibacter styleae*	10 (G), 12 (S, U)	NR_181360.1	99.2
*Pseudoalteomonas citrea*	10 (U)	KX453254.1	98.6
*Pseudoalteromonas* sp.	10 (G)	PQ670272.1	98.5
*Pseudomonas* sp.	3 (G, U)	JQ723717.1	99.8
*Psychrobacter* sp.	10 (U)	EF190337.1	95.1
*Rathayibacter* sp.	12 (S)	PQ596301.1	98.8
*Roseibium aggregatum*	3 (U)	OP355453.1	98.2
*Ruegeria* sp.	10 (S)	KY770077.1	99.8
*Tenacibaculum* sp.	12 (S, G)	KU560487.1	100
*Tritonnibacter scottomollicae*	10 (S)	NR_042675.1	99.6
*Vibrio alginolyticus*	4 (S)	MN945277.1	99.4
*Vibrio* sp.	3 (G), 4 (G)	OQ553684.1	98.6

**Table 3:** Species, month (3 = third month, October; 4 = forth month, November; 10 = tenth month, May; 12 = twelfth month, July), condition (S: suspended, G: over gravel; U: under gravel), accession number, and percentage of identity are reported for PCL.

Species	Sampling Month	Accession	Identity (%)
*Alkalihalobacillus algicola*	3 (U)	OR262827.1	99.3
*Alkalihalobacillus hemicentroti*	10 (G)	MF197940.1	84.2
*Alteromonas* sp.	12 (S, G)	KR269635.1	98.3
*Bacillus* sp.	4 (G), 12 (U)	KC815809.1	96.6
*Glaciecola* sp.	4 (U)	JX310209.1	95.4
*Halomonas* sp.	3 (G)	PP446495.1	86.6
*Halopseudomonas gallaeciensis*	4 (U)	OR225156.1	94.4
*Halopseudomonas* sp.	3 (U)	OQ780586.1	93.2
*Kocuria rosea*	3 (S)	OP268600.0	94.4
*Magnetococcus* sp.	12 (U)	ON340531.1	88.2
*Marinobacter algicola*	3 (S)	LT600680.1	98
*Marinobacter sediminum*	4 (U)	NR_029028.1	99
*Marinobacter similis*	4 (U)	NR_178677.1	99
*Marinobacter* sp.	3 (G)	AP028070.1	96.6
*Paenihalocynthiibacter styelae*	10 (U)	NR_181360.1	99.4
*Paraglaciecola* sp.	4 (S, U)	OQ119896.1	95.7
*Photobacterium* sp.	12 (G)	JX134425.1	99.5
*Pseudoalteromoas* sp.	4 (U), 3 (U)	FJ169986.1	90.4
*Pseudomonas* sp.	3 (S, G, U), 4 (G, U)	EU249981.1	100
*Roseovarius* sp.	12 (G)	MF948949.1	95.1
*Rossellomorea vietnamensis*	4 (G)	PQ629588.1	89.2
*Ruegeria* sp.	4 (G), 10 (S)	KY770246.1	100
*Sulfitobacter* sp.	3 (S)	AJ534212.1	91.8
*Tenacibaculum xiamenense*	12 (S)	NR_109729.1	97.2
*Thalassospira permensis*	12 (U)	KX027355.1	96.8
*Tritonnibacter scottomollicae*	4 (G)	NR_042675.1	99.4

**Table 4:** Species, month (3 = third month, October; 4 = forth month, November; 10 = tenth month, May; 12 = twelfth month, July), condition (S: suspended; G: over gravel; U: under gravel), accession number, and percentage of identity are reported for PHB films.

Species	Month (Condition)	Accession	Identity (%)
*Enterobacter* sp.	10 (U)	OK035565.1	94.6
*Halomonas* sp.	3 (S)	AB305244.1	97.7
*Labrenzia* sp.	12 (S)	MG025898.1	100
*Marinobacter algicola*	3 (U), 10 (S, G)	LT600552.1	99.1
*Marinobacter* sp.	3 (S, U), 12 (S)	PQ278257.1	97.5
*Pseudoalterommonas prydzensis*	3 (G)	KP236351.1	94.4
*Pseudoalteromonas citrea*	10 (G)	KX453254.1	95.9
*Pseudoalteromonas* sp.	10 (U)	KY272049.1	99.7
*Pseudomonas aeruginosa*	10 (U)	CP007224.1	94.6
*Pseudomonas* sp.	3 (G), 4 (U)	AM403176.1	94
*Roseibium aggregatum*	12 (S)	OQ553960.1	99.2
*Thalassospira permensis*	10 (U), 12 (U)	KX027355.1	99.4
*Tritonibacter litoralis*	12 (G)	NR_180841.1	99.7
*Vibrio alginolyticus*	10 (U)	PQ670272.1	94.6
*Vibrio coraliilyticus*	4 (G), 12 (S)	MW828508.1	99.7
*Vibrio* sp.	4 (U)	CP155566.1	95.9

**Table 5:** Species, month (3 = third month, October; 4 = forth month, November; 10 = tenth month, May; 12 = twelfth month, July), condition (S: suspended; G: over gravel; U: under gravel), accession number, percentage of identity, and number of colonies are reported for PLA films.

Species	Month (Condition)	Accession	Identity (%)
*Alteromonas australica*	3 (S, G), 12 (S)	OP342934.1	99.4
*Alteromonas confluentis*	12 (S)	OR262787.1	97.2
*Alteromonas macleodii*	4 (G, U)	KM041224.1	96.7
*Alteromonas* sp.	3 (U), 4 (S)	MG696193.1	92.4
*Bacillus aquimaris*	3 (U)	MZ430464.1	96.7
*Bacillus hwajinpoensis*	12 (S)	KF933706.1	98.3
*Cyanobium* sp.	3 (S)	HM217050.1	97.6
*Glaciecola* sp.	10 (S), 12 (S)	JX310209.1	99.3
*Halomonas* sp.	10 (G, U), 12 (S)	KF201593.1	99.7
*Halomonas taenennsis*	3 (G)	MK063863.1	96.8
*Idiomarina ioihiensis*	3 (S)	KP860601.1	99.7
*Idiomarina ramblicola*	3 (S)	MK063828.1	99.7
*Leisingera caerulea*	10 (G)	MW422659.1	97.8
*Leisingera* sp.	10 (U), 12 (U)	KU554489.1	100
*Marinobacter* sp.	3 (U)	MF401328.1	98.6
*Microbulbifer* sp.	3 (S)	KM362894.1	99.1
*Paraglaciecola* sp.	10 (S)	PQ856828.1	98.9
*Phaebacyer* sp.	10 (G, U)	HM031995.1	99.4
*Pseudoalteromonas citrea*	10 (G, U)	KX453254.1	99.3
*Pseudoalteromonas* sp.	10 (G, U)	PQ670272.1	99.1
*Pseudomonas* sp.	4 (S)	KF786975.1	89.1
*Psychrobacter submarinus*	10 (S)	KF424825.1	97.8
*Ruegeria atlantica*	12 (U)	JX463483.1	97.5
*Sulfitobacter dubius*	12 (U)	MZ292263.1	97.4
*Sutcliffiella horikoshi*	12 (G)	OQ560489.1	99.1
*Tenacibaculum xiamenense*	12 (U)	NR_109729.1	95.4

## Data Availability

The original contributions presented in this study are included in the article/Supplementary Material. Further inquiries can be directed to the corresponding authors.

## Supplementary Materials

The following supporting information can be downloaded at: https://www.mdpi.com/article/10.3390/microorganisms13030609/s1, Figure S1: Photo of one tank of the experimental mesocosms, consisting of fifteen experimental tanks. The bottom of the tank was covered with natural gravel (coralline sand, grain size 0.4–0.8, Arena Silex, Manufacturas Gre, S.A.). Three experimental conditions where the polymers were placed: (a) suspended in sea water, immersed about 10 cm from the surface of the water; (b) laying over the gravel; (c) under the gravel. Figure S2: Panel A shows common or specific bacteria when considering different positions of the polymers in the mesocosm (S = suspended; U = under gravel; G = gravel); panel B shows common or specific bacteria when considering different timepoints (3 = October; 4 = November; 10 = May; 12 = July); panel C shows common or specific bacteria when considering different plastics. Table S1: Main nutrients (NH_4_, NO_2_, NO_3_, PO_4_, reported as mg/L) were measured twice a month during all experiments. Data are reported as mean average (±standard deviation) among the 15 tanks. Table S2: Physical parameters (temperature in °C, pH, oxygen as mg/L, salinity as PSU) were measured three times a week during all experiments. Data are reported as mean average (± standard deviations) among the 15 tanks. Table S3: Composition of Zobell Marine Agar, reporting each compound and concentration in grams (g)/litres (L).

## References

[1] Van Eygen E., Feketitsch J., Laner D., Rechberger H., Fellner J. (2017). Comprehensive analysis and quantification of national plastic flows: The case of Austria. Resour. Conserv. Recycl..

[2] Filho W.L., Salvia A.L., Bonoli A., Saari U.A., Voronova V., Klõga M., Kumbhar S.S., Olszewski K., De Quevedo D.M., Barbir J. (2021). An assessment of attitudes towards plastics and bioplastics in Europe. Sci. Total Environ..

[3] Adane L., Muleta D. (2011). Survey on the usage of plastic bags, their disposal and adverse impacts on environment: A case study in Jimma City, Southwestern Ethiopia. J. Toxicol. Environ. Health Sci..

[4] North E.J., Halden R.U. (2013). Plastics and environmental health: The road ahead. Rev. Environ. Health.

[5] Thakur S., Chaudhary J., Sharma B., Verma A., Tamulevicius S., Thakur V.K. (2018). Sustainability of bioplastics: Opportunities and challenges. Curr. Opin. Green Sustain. Chem..

[6] Palazzo L., Coppa S., Camedda A., Cocca M., De Falco F., Vianello A., Massaro G., de Lucia G.A. (2021). A novel approach based on multiple fish species and water column compartments in assessing vertical microlitter distribution and composition. Environ. Pollut..

[7] Santonicola S., Volgare M., Rossi F., Castaldo R., Cocca M., Colavita G. (2024). Detection of fibrous microplastics and natural in fish species (*Engraulis encrasicolus*, *Mullus barbatus*, and *Merluccius merliccius*) for human consumption from the Tyrrhennian sea. Chemosphere.

[8] Abioye A.A., Obuekwe C., Fasanmi O., Oluwadare O., Abioye O.P., Afolalu S.A., Akinlabi S.A., Bolu C.A. (2019). Investigation of biodegradation speed and biodegradability of polyethylene and *Manihot Esculenta* starch blends. J. Ecol. Eng..

[9] Sharma B., Jain P. (2020). Deciphering the advances in bioaugmentation of plastic wastes. J. Clean. Prod..

[10] Yanti N.A., Sembiring L., Margino S., Ahmad S.W. (2021). Bacterial Production of Poly-b-hydroxybutyrate (PHB): Converting Starch into Bioplastics. Bioplastics for Sustainable Development.

[11] Bano R., Kuddus K., Zaheer M.R., Zia M. (2017). Mohammed Microbial enxymatic degradation of biodegradable plastics. Curr. Pharm. Biotechnol..

[12] Gross R.A., Kalra B. (2002). Biodegradable polymers for the environment. Science.

[13] Yu J., Chen L.X.L. (2008). The greenhouse gas emissions and fossil energy requirement of bioplastics from cradle to gate of a biomass refinery. Environ. Sci. Technol..

[14] Agarwal S. (2020). Biodegradable Polymers: Present Opportunities and Challenges in Providing a Microplastic-Free Environment. Macromol. Chem. Phys..

[15] Viel T., Cocca M., Manfra L., Caramiello D., Libralato G., Zupo V., Costantini M. (2023). Effects of biodegradable-based microplastics in *Paracentrotus lividus* Lmk embryos: Morphological and gene expression analysis. Environ. Pollut..

[16] Manfra L., Marengo V., Libralato G., Costantini M., De Falco F., Cocca M. (2021). Biodegradable polymers: A real opportunity to solve marine plastic pollution?. J. Hazard. Mater..

[17] Viel T., Cocca M., Esposito R., Amato A., Russo T., Di Cosmo A., Polese G., Manfra L., Libralato G., Zupo V. (2024). Effect of biodegradable polymers upon grazing activity of the sea urchin *Paracentrotus lividus* (Lmk) revealed by morphological, histological and molecular analyses. Sci. Total Environ..

[18] Samir A., Ashour F.H., Hakim A.A.A., Bassyouni M. (2022). Recent advances in biodegradable polymers for sustainable applications. npj Mater. Degrad..

[19] Danso D., Chow J., Streita W.R. (2019). Plastics: Environmental and biotechnological perspectives on microbial degradation. Appl. Environ. Microbiol..

[20] Zeenat, Elahi A., Bukhari D.A., Shamim S., Rehman A. (2021). Plastics degradation by microbes: A sustainable approach. J. King Saud Univ.-Sci..

[21] Koh J., Bairoliya S., Salta M., Cho Z.T., Fong J., Neo M.L., Cragg S., Cao B. (2023). Sediment-driven plastisphere community assembly on plastic debris in tropical coastal and marine environments. Environ. Int..

[22] Zettler E.R., Mincer T.J., Amaral-Zettler L.A. (2013). Life in the “plastisphere”: Microbial communities on plastic marine debris. Environ. Sci. Technol..

[23] Wright R.J., Erni-Cassola G., Zadjelovic V., Latva M., Christie-Oleza J.A. (2020). Marine plastic debris: A new surface for microbial colonization. Environ. Sci. Technol..

[24] Zhu B., Wang D., Wei N. (2022). Enzyme discovery and engineering for sustainable plastic recycling. Trends Biotechnol..

[25] Zhang S., Wu H., Hou J. (2023). Progress on the effects of microplastics on aquatic crustaceans: A review. Int. J. Mol. Sci..

[26] Kim J., Park S., Jung S., Yun H., Choi K., Heo G., Jin H.J., Park S., Kwak H.W. (2023). Biodegradation behavior of polybutylene succinate (PBS) fishing gear in marine sedimentary environments for ghost fishing prevention. Polym. Degrad. Stab..

[27] Lu B., Wang G.X., Huang D., Ren Z.L., Wang X.W., Wang P.L., Zhen Z.C., Zhang W., Ji J.H. (2018). Comparison of PCL degradation in different aquatic environments: Effects of bacteria and inorganic salts. Polym. Degrad. Stab..

[28] Jeon Y., Jin H.J., Kong Y., Cha H.G., Lee B.W., Yu K., Yi B., Kim H.T., Joo J.C., Yang Y.H. (2023). Poly(3-hydroxybutyrate) degradation by *Bacillus infantis* sp. isolated from soil and identification of phaZ and bdhA expressing PHB depolymerase. J. Microbiol. Biotechnol..

[29] Bubpachat T., Sombatsompop N., Prapagdee B. (2018). Isolation and role of polylactic acid-degrading bacteria on degrading enzymes productions and PLA biodegradability at mesophilic conditions. Polym. Degrad. Stab..

[30] Wang S., Shi W., Huang Z., Zhou N., Xie Y., Tang Y., Hu F., Liu G., ZHeng H. (2022). Complete digestion/biodegradation of polystyrene microplastics by greater wax moth (*Galleria mellonella*) larvae: Direct in vivo evidence, gut microbiota independence, and potential metabolic pathways. J. Hazard. Mater..

[31] Joshi G., Goswami P., Verma P., Prakash G., Simon P., Vinithkumar N.V., Dharani G. (2022). Unraveling the plastic degradation potentials of the plastisphere-associated marine bacterial consortium as a key player for the low-density polyethylene degradation. J. Hazard. Mater..

[32] Jaduan J.S., Bansal S., Sonthalia A., Rai A.K., Singh S.P. (2022). Biodegradation of plastics for sustainable environment. Bioresour. Technol..

[33] Li X., Liu X., Zhang J., Chen F., Khalid M., Ye J., Romantschuk M., Hui N. (2024). Hydrolase and plastic-degrading microbiota explain degradation of polyethylene terephthalate microplastics during high-temperature composting. Bioresour. Technol..

[34] Lv S., Li Y., Zhao S., Shao Z. (2024). Biodegradation of typical plastics: From microbial diversity to metabolic mechanisms. Int. J. Mol. Sci..

[35] Ruocco N., Bertocci I., Munari M., Musco L., Caramiello D., Danovaro R., Zupo V., Costantini M. (2020). Morphological and molecular responses of the sea urchin *Paracentrotus lividus* to highly contaminated marine sediments: The case study of Bagnoli-Coroglio brownfield (Mediterranean Sea). Mar. Environ. Res..

[36] Yurkov V.V., van Gemerden H. (1993). Impact of light/dark regimen on growth rate, biomass formation and bacteriochlorophyll synthesis in *Erythromicrobium hydrolyticum*. Arch. Microbiol..

[37] Liu Y., Lim C.K., Shen Z., Lee P.K.H., Nah T. (2023). Effects of pH and light exposure on the survival of bacteria and their ability to biodegrade organic compounds in clouds: Implications for microbial activity in acidic cloud water. Atmos. Chem. Phys..

[38] Grigioni S., Boucher-Rodoni R., Demarta A., Tonolla M., Peduzzi R. (1999). Phylogenetic characterisation of bacterial symbionts in the accessory nidamental glands of the sepioid *Sepia officinalis* (Cephalopoda: Decapoda). Mar. Biol..

[39] Camacho C., Coulouris G., Avagyan V., Ma N., Papadopoulos J., Bealer K., Madden L.T. (2009). BLAST+: Architecture and applications. BMC Bioinform..

[40] Van Rossum G., Drake F.L. (2009). Introduction to Python 3: Python Documentation Manual Part 1.

[41] Pedregosa F., Varoquaux G., Gramfort A., Michel V., Thirion B. (2011). Scikit-learn: Machine learning in Python. J. Mach. Learn. Res..

[42] Halford M.P. https://github.com/MaxHalford/prince.

[43] Waskom M. (2021). Seaborn: Statistical Data Visualization. J. Open Source Softw..

[44] De Falco F., Avolio R., Errico M.E., Di Pace E., Avella M., Cocca M., Gentile G. (2021). Comparison of biodegradable polyesters degradation behavior in sand. J. Hazard. Mater..

[45] Frontier S. (1976). Étude de la décroissance des valeurs propres dans une analyse en composantes principales: Comparaison avec le modd’le du bâton brisé. J. Exp. Mar. Biol. Ecol..

[46] Carpenter E.J., Anderson S.J., Harvey J.R., Miklas H.P., Peck B.B. (1972). Polystyrene Spherules in coastal Waters. Science.

[47] Carpenter E.J., Smith K.L.J. (1972). Plastics on the Sargasso Sea Surface. Science.

[48] Gregory M.R. (2009). Environmental implications of plastic debris in marine settings- entanglement, ingestion, smothering, hangers-on, hitch-hiking and alien invasions. Philos. Trans. R. Soc. B Biol. Sci..

[49] Roager L., Sonnenschein E.C. (2019). Bacterial Candidates for Colonization and Degradation of Marine Plastic Debris. Environ. Sci. Technol..

[50] He M., Hsu Y.I., Uyama H. (2024). Superior sequence-controlled poly(L-lactide)-based bioplastic with tunable seawater biodegradation. J. Hazard. Mater..

[51] Dang H., Li T., Chen M., Huang G. (2008). Cross-ocean distribution of *Rhodobacterales* bacteria as primary surface colonizers in temperate coastal marine waters. Appl. Environ. Microbiol..

[52] Balasubramanian V., Natarajan K., Hemambika B., Ramesh N., Sumathi C.S., Kottaimuthu R., Rajesh Kannan V. (2010). High-density polyethylene (HDPE)-degrading potential bacteria from marine ecosystem of Gulf of Mannar, India. Lett. Appl. Microbiol..

[53] Danko A.S., Luo M., Bagwell C.E., Brigmon R.L., Freedman D.L. (2004). Involvement of linear plasmids in aerobic biodegradation of vinyl chloride. Appl. Environ. Microbiol..

[54] Ghosh S.K., Pal S., Ray S. (2013). Study of microbes having potentiality for biodegradation of plastics. Environ. Sci. Pollut. Res. Int..

[55] Kumar Sen S., Raut S. (2015). Microbial degradation of low density polyethylene (LDPE): A review. J. Environ. Chem. Eng..

[56] Mohan A.J., Sekhar V.C., Bhaskar T., Nampoothiri K.M. (2016). Microbial assisted High Impact Polystyrene (HIPS) degradation. Bioresour. Technol..

[57] Shah A.A., Hasan F., Hameed A., Ahmed S. (2008). Biological degradation of plastics: A comprehensive review. Biotechnol. Adv..

[58] Shimpi N., Borane M., Mishra S., Kadam M. (2012). Biodegradation of polystyrene (PS)-poly(lactic acid) (PLA) nanocomposites using *Pseudomonas aeruginosa*. Macromol. Res..

[59] Suzuki M., Tachibana Y., Oba K., Takizawa R., Kasuya K. (2018). ichi Microbial degradation of poly(ε-caprolactone) in a coastal environment. Polym. Degrad. Stab..

[60] Oberbeckmann S., Loeder M.G.J., Gerdts G., Osborn M.A. (2014). Spatial and seasonal variation in diversity and structure of microbial biofilms on marine plastics in Northern European waters. FEMS Microbiol. Ecol..

[61] Oberbeckmann S., Osborn A.M., Duhaime M.B. (2016). Microbes on a bottle: Substrate, season and geography influence community composition of microbes colonizing marine plastic debris. PLoS ONE.

[62] Oberbeckmann S., Kreikemeyer B., Labrenz M. (2018). Environmental factors support the formation of specific bacterial assemblages on microplastics. Front. Microbiol..

[63] Bryant J.A., Clemente T.M., Viviani D.A., Fong A.A., Thomas K.A., Kemp P., Karl D.M., White A.E., DeLong E.F. (2016). Diversity and Activity of Communities Inhabiting Plastic Debris in the North Pacific Gyre. mSystems.

[64] Frère L., Maignien L., Chalopin M., Huvet A., Rinnert E., Morrison H., Kerninon S., Cassone A.L., Lambert C., Reveillaud J. (2018). Microplastic bacterial communities in the Bay of Brest: Influence of polymer type and size. Environ. Pollut..

[65] Syranidou E., Karkanorachaki K., Amorotti F., Franchini M., Repouskou E., Kaliva M., Vamvakaki M., Kolvenbach B., Fava F., Corvini P.F.X. (2017). Biodegradation of weathered polystyrene films in seawater microcosms. Sci. Rep..

[66] Pollet T., Berdjeb L., Garnier C., Durrieu G., Le Poupon C., Misson B., Briand J.F. (2018). Prokaryotic community successions and interactions in marine biofilms: The key role of Flavobacteriia. FEMS Microbiol. Ecol..

[67] Butbunchu N., Pathom-Aree W. (2019). Actinobacteria as Promising Candidate for Polylactic Acid Type Bioplastic Degradation. Front. Microbiol..

[68] Prasad R., Kumar V., Singh J., Prakash C., Editors U. (2020). Recent Developments in Microbial Technologies.

[69] Dussud C., Meistertzheim A.L., Conan P., Pujo-Pay M., George M., Fabre P., Coudane J., Higgs P., Elineau A., Pedrotti M.L. (2018). Evidence of niche partitioning among bacteria living on plastics, organic particles and surrounding seawaters. Environ. Pollut..

[70] Didier D., Anne M., Alexandra T.H. (2017). Plastics in the North Atlantic garbage patch: A boat-microbe for hitchhikers and plastic degraders. Sci. Total Environ..

[71] Kimura Y., Fukuda Y., Otsu R., Yu J., Mino S., Misawa S., Maruyama S., Ikeda Y., Miyamachi R., Noguchi H. (2023). A lesson from polybutylene succinate plastisphere to the discovery of novel plastic degrading enzyme genes in marine vibrios. Environ. Microbiol..

[72] Kim S.H., Cho J.Y., Cho D.H., Jung H.J., Kim B.C., Bhatia S.K., Park S.H., Park K., Yang Y.H. (2022). Acceleration of Polybutylene Succinate Biodegradation by *Terribacillus* sp. JY49 isolated from a marine environment. Polymers.

[73] Suzuki M., Ishii S., Gonda K., Kashima H., Suzuki S., Uematsu K., Arai T., Tachibana Y., Iwata T., Kasuya K. (2022). Marine Biodegradation Mechanism of Biodegradable Plastics Revealed by Plastisphere Analysis. Res. Sq..

[74] Soulenthone P., Suzuki M., Tachibana Y., Furukori M., Saito T., Kawamura R., Bankole P.O., Kasuya K. (2025). ichi *Halopseudomonas* sp. MFKK-1: A marine-derived bacterium capable of degrading poly(butylene succinate-co-adipate), poly(ε-caprolactone), and poly(butylene adipate-co-terephthalate) in marine ecosystems. Polym. Degrad. Stab..

[75] Zampolli J., Vezzini D., Brocca S., Di Gennaro P. (2023). Insights into the biodegradation of polycaprolactone through genomic analysis of two plastic-degrading *Rhodococcus* bacteria. Front. Microbiol..

[76] Nawaz A., Hasan F., Shah A.A. (2015). Degradation of poly(ε-caprolactone) (PCL) by a newly isolated *Brevundimonas* sp. strain MRL-AN1 from soil. FEMS Microbiol. Lett..

[77] Pan H., Yu T., Zheng Y., Ma H., Shan J., Yi X., Liu Y., Zhan J., Wang W., Zhou H. (2024). Isolation, characteristics, and poly(butylene adipate-co-terephthalate) (PBAT) degradation mechanism of a marine bacteria *Roseibium aggregatum* ZY-1. Mar. Pollut. Bull..

[78] Gao R., Sun C. (2020). A marine bacterial community that degrades poly(ethylene terephthalate) and polyethylene. bioRxiv.

[79] Yao Z., Seong H.J., Jang Y.S. (2022). Degradation of low density polyethylene by Bacillus species. Appl. Biol. Chem..

[80] Yang H.S., Yoon J.S., Kim M.N. (2005). Dependence of biodegradability of plastics in compost on the shape of specimens. Polym. Degrad. Stab..

[81] Chinaglia S., Tosin M., Degli-Innocenti F. (2018). Biodegradation rate of biodegradable plastics at molecular level. Polym. Degrad. Stab..

[82] Du Y., Liu X., Dong X., Yin Z. (2022). A review on marine plastisphere: Biodiversity, formation, and role in degradation. Comput. Struct. Biotechnol. J..

[83] Al Hosni A.S., Pittman J.K., Robson G.D. (2019). Microbial degradation of four biodegradable polymers in soil and compost demonstrating polycaprolactone as an ideal compostable plastic. Waste Magagement.

[84] Rafiq M., Hassan N., Rehman M., Hayat M., Nadeem G., Hassan F., Iqbal N., Ali H., Zada S., Kang Y. (2023). Challenges and approaches of culturing the unculturable archaea. Biology.

[85] Silva R.R.A., Marques C.S., Arruda T.R., Teixeira S.C., de Oliveira T.V. (2023). Biodegradation of polymers: Stages, measurement, standards and prospects. Macromol.

[86] Cao Z., Yan W., Ding M., Yuan Y. (2022). Construction of microbial consortia for microbial degradation of complex compounds. Front. Bioeng. Biotechnol..

[87] Su T., Zhang T., Liu P., Bian J., Zheng Y., Yuan Y., Li Q., Liang Q., Qi Q. (2023). Biodegradation of polyurethane by the microbial consortia enriched from landfill. Appl. Microbiol. Biotechnol..

[88] Martín-González D., de la Fuente Tagarro C., De Lucas A., Bordel S., Santos-Beneit F. (2024). Genetic modifications in bacteria for the degradation of synthetic polymers: A review. Int. J. Mol. Sci..

