# Supplementary material for: Isolation and Identification of Bacterial Strains Colonizing the Surface of Biodegradable Polymers
Source: Microorganisms. 2025 Mar 6;13(3):609. doi: 10.3390/microorganisms13030609 (PMC11944904; doi:10.3390/microorganisms13030609)
Supplement: Supplementary file 1 [file microorganisms-13-00609-s001.zip › microorganisms-3490322-supplementary.pdf]

Isolation and Identification of Bacterial Strains Colonizing the Surface of Biodegradable Polymers

Roberta Esposito [1,†], Serena Federico [1,†], Amalia Amato [1,2], Thomas Viel [1,2,3], Davide Caramiello [4], Alberto Macina [4], Marco Miralto [5], Luca Ambrosino [5], Maria Luisa Chiusano [5], Mariacristina Cocca [3], Loredana Manfra [1,6], Giovanni Libralato [1,2], Valerio Zupo [7,*,‡] and Maria Costantini [1,*,‡]

[1] Department of Ecosustainable Marine Biotechnology, Stazione Zoologica Anton Dohrn, Villa Comunale, 80121 Naples, Italy; roberta.esposito@szn.it (R.E.); serena.federico@szn.it (S.F.); amalia.amato@szn.it (A.A.); thomas.viel7@gmail.com (T.V.); loredana.manfra@isprambiente.it (L.M.); giovanni.libralato@unina.it (G.L.)
[2] Department of Biology, University of Naples Federico II, Via Cinthia 26, 80126 Napoli, Italy
[3] Institute of Polymers, Composites and Biomaterials, National Research Council of Italy, Via Campi Flegri, 34, Pozzuoli, 80078 Naples, Italy; mariacristina.cocca@ipcb.cnr.it
[4] Department of Marine Animal Conservation and Public Engagement, Stazione Zoologica Anton Dohrn, Villa Comunale, 1, 80121 Naples, Italy; davide.caramiello@szn.it (D.C.); alberto.macina@szn.it (A.M.)
[5] Department of Research Infrastructures for Marine Biological Resources, Stazione Zoologica Anton Dohrn, Villa Comunale, 80121 Napoli, Italy; marco.miralto@szn.it (M.M.); luca.ambrosino@szn.it (L.A.); mcosta@szn.it (M.L.C.)
[6] Institute for Environmental Protection and Research (ISPRA), Via Vitaliano Brancati 48, 00144 Rome, Italy
[7] Department of Ecosustainable Marine Biotechnology, Stazione Zoologica Anton Dohrn, Ischia Marine Centre, Ischia, 80077 Naples, Italy
* Correspondence: vzupo@szn.it (V.Z.); maria.costantini@szn.it (M.C.)
† These authors contributed equally to this work.
‡ These authors contributed equally to this work.

Figure S1. Photo of one tank of the experimental mesocosms, consisting of fifteen experimental tanks. The bottom of tank was covered with natural gravel (coralline sand, grain size 0.4–0.8, Arena Silex, Manufacturas Gre, S.A.) and the three experimental conditions where the polymers were placed: a) suspended in the sea water, immersed at about 10 cm from the surface of the water, b) laying over the gravel; c) under the gravel.

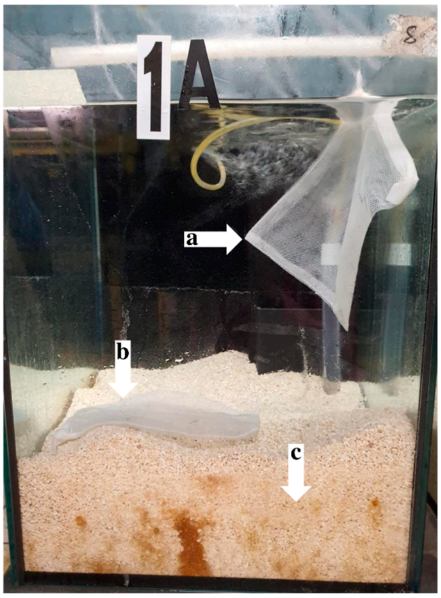

Figure S2. Panel A shows common or specific bacteria when considering different positions of the polymers in the mesocosm (S = suspended; U = under gravel; G = gravel); panel B shows common or specific bacteria when considering different timepoints (3 = October; 4 = November; 10 = May; 12 = July); panel C shows common or specific bacteria when considering different plastics.

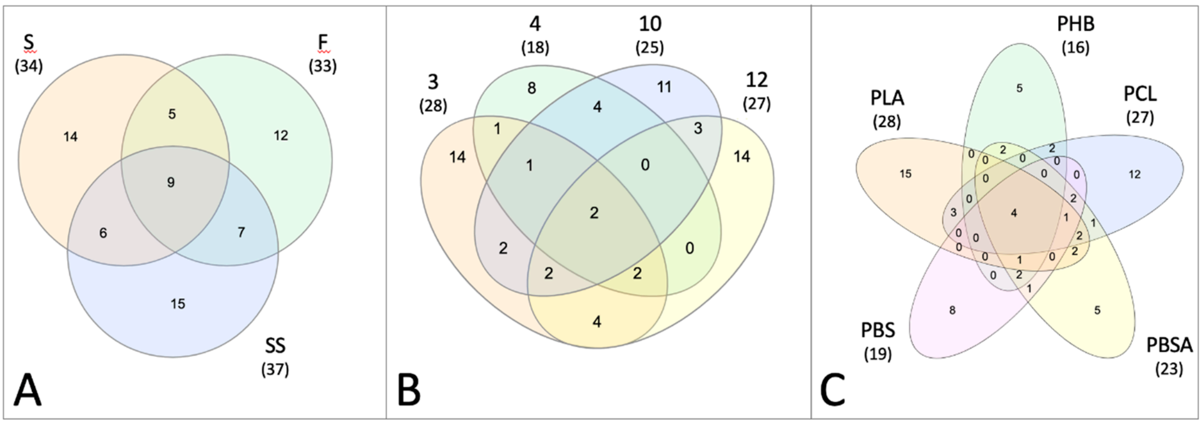

Table S1. Main nutrients (NH_4, NO_2, NO_3, PO_4, reported as mg/L; twice a monthwere measured during all the experiments. Data are reported as mean average (± standard deviation) among the 15 tanks.

	**NH_4**	**NO_2**	**NO_3**	**PO_4**
**August**	0.000 ± 0.000	0.005 ±0.002	1.653 ±0.642	0.255 ± 0.143
**September**	0.003 ± 0.005	0.006 ± 0.004	1.760 ± 0.855	0.313 ± 0.145
**October**	0.000 ± 0.000	0.006 ±0.003	1.240 ±0.495	0.224 ± 0.109
**November**	0.007 ± 0.013	0.005 ± 0.001	1.080 ± 0.629	0.182 ±0.066
**December**	0.007 ± 0.012	0.006 ±0.003	1.008 ±0.470	0.154 ±0.085
**January**	0.002 ±0.004	0.013 ±0.002	1.117 ±0.463	0.147 ± 0.044
**February**	0.000 ± 0.000	0.007 ± 0.003	1.175 ± 0.698	0.141 ± 0.52
**March**	0.000 ± 0.000	0.006 ± 0.003	1.075 ± 0.634	0.150 ± 0.086
**April**	0.005 ± 0.010	0.006 ± 0.002	1.083 ± 0.595	0.309 ± 0.337
**May**	0.000 ± 0.009	0.005 ± 0.002	1.483 ± 0.585	0.208 ± 0.062
**June**	0.005 ± 0.010	0.005 ± 0.002	1.283 ± 0.374	0.167 ± 0.082
**July**	0.000 ± 0.000	0.002 ± 0.003	1.067 ± 0.612	0.102 ± 0.050

Table S2. Physical parameters (temperature in °C, pH, oxygen as mg/L, salinity as PSU; three times a week) were measured during all the experiments. Data are reported as mean average (± standard deviations) among the 15 tanks.

	**Temperature**	**pH**	**Oxygen**	**Salinity**
**August**	26.98 ± 0.80	8.11 ± 0.19	6.90 ± 0.24	37.89 ± 0.35
**September**	25.20 ± 0.61	7.93 ± 0.21	7.22 ± 0.10	38.12 ± 0.47
**October**	21.43 ± 1.56	7.81 ± 0.17	7.71 ± 0.22	38.43 ± 0.66
**November**	20.04 ± 0.89	7.85 ± 0.07	7.86 ± 0.11	38.60 ± 0.62
**December**	23.15 ± 0.72	7.90 ± 0.06	7.40 ± 0.13	38.30 ± 0.89
**January**	16.13 ± 0.84	7.98 ± 0.08	8.06 ± 0.53	37.33 ± 0.91
**February**	16.10 ± 0.52	8.10 ± 0.07	8.54 ± 0.10	37.98 ± 0.92
**March**	15.72 ± 0.77	8.09 ± 0.08	8.61 ± 0.12	38.16 ± 0.72
**April**	17.43 ± 0.97	8.10 ± 0.08	8.28 ± 0.17	38.50 ± 0.45
**May**	21.62 ±1.82	8.09 ± 0.07	7.70 ± 0.25	38.69 ± 0.31
**June**	25.32 ± 1.40	8.04 ± 0.06	7.05 ± 0.18	38.60 ± 0.50
**July**	25.59 ± 0.46	7.96 ± 0.06	7.17 ± 0.06	38.43 ± 1.88

Table S3. Composition of the Zobell Marine Agar. reporting each compound and concentration in grams (g) / litres (L).

**Compound**	**Concentration (g/L)**
Peptone	5.00
Yeast extract	1.00
Ferric citrate	0.10
Sodium Chloride	19.45
Magnesium Chloride	8.80
Sodium sulphate	3.24
Calcium chloride anhydrous	1.80
Potassium chloride	0.55
Sodium bicarbonate	0.16
Potassium bromide	0.08
Strontium chloride	0.034
Boric acid	0.022
Sodium silicate	0.004
Sodium fluorate	0.0024
Ammonium nitrate	0.0016
Disodium hydrogen phosphate	0.008
Agar	15.00
